# T cell subsets: an immunological biomarker to predict progression to clinical arthritis in ACPA-positive individuals

**DOI:** 10.1136/annrheumdis-2015-207991

**Published:** 2015-12-01

**Authors:** L Hunt, E M Hensor, J Nam, A N Burska, R Parmar, P Emery, F Ponchel

**Affiliations:** 1 Leeds Institute of Rheumatic and Musculoskeletal Medicine, University of Leeds, Chapel Allerton Hospital, Leeds, UK; 2 NIHR Leeds Musculoskeletal Biomedical Research Unit, Leeds Teaching Hospitals NHS Trust, Leeds, UK

**Keywords:** Arthritis, Synovitis, T Cells

## Abstract

**Objectives:**

Anticitrullinated protein antibody (ACPA)+ individuals with non-specific musculoskeletal symptoms are at risk of inflammatory arthritis (IA). This study aims to demonstrate the predictive value of T cell subset quantification for progression towards IA and compare it with previously identified clinical predictors of progression.

**Methods:**

103 ACPA+ individuals without clinical synovitis were observed 3-monthly for 12 months and then as clinically indicated. The end point was the development of IA. Naïve, regulatory T cells (Treg) and inflammation related cells (IRCs) were quantified by flow cytometry. Areas under the ROC curve (AUC) were calculated. Adjusted logistic regressions and Cox proportional hazards models for time to progression to IA were constructed.

**Results:**

Compared with healthy controls (age adjusted where appropriate), ACPA+ individuals demonstrated reduced naïve (22.1% of subjects) and Treg (35.8%) frequencies and elevated IRC (29.5%). Of the 103 subjects, 48(46.6%) progressed. Individually, T cell subsets were weakly predictive (AUC between 0.63 and 0.66), although the presence of 2 T cell abnormalities had high specificity. Three models were compared: model-1 used T cell subsets only, model-2 used previously published clinical parameters, model-3 combined clinical data and T cell data. Model-3 performed the best (AUC 0.79 (95% CI 0.70 to 0.89)) compared with model-1 (0.75 (0.65 to 0.86)) and particularly with model-2 (0.62 (0.54 to 0.76)) demonstrating the added value of T cell subsets. Time to progression differed significantly between high-risk, moderate-risk and low-risk groups from model-3 (p=0.001, median 15.4 months, 25.8 months and 63.4 months, respectively).

**Conclusions:**

T cell subset dysregulation in ACPA+ individuals predates the onset of IA, predicts the risk and faster progression to IA, with added value over previously published clinical predictors of progression.

## Introduction

Over recent years our understanding of the immune pathways and interactions involved in the pathogenesis of rheumatoid arthritis (RA) has evolved substantially. This has had a notable impact on drug development targeting specific pathways. Early RA clinical trials have aided the translation of findings and resulted in a vast body of evidence supporting early diagnosis and immediate treatment to improve outcomes of patients with RA.^1–4^ However despite early intervention at RA diagnosis, a proportion of individuals fails conventional therapy and continues with immune dysregulation and active inflammation.^5–7^ This has led investigators to focus on identifying disease at its earliest stage.^8^ By identifying individuals at a higher risk of future RA, it is hoped that outcomes can be improved.

Several groups including our own have reported on cohorts at high risk to RA.^9–15^ The most notable of these are individuals with RA-associated anticitrullinated protein antibody (ACPA) autoantibodies and musculoskeletal pain. However, autoantibodies alone are not sufficient to predict progression to inflammatory arthritis (IA) with only 50% overall progression over 4 years.^14^ In recent years there has been increased interest in the identification of biomarkers that assist the prediction of disease onset in such cohorts.^16–26^ The ability to risk stratify individuals is an attractive option particularly in light of current strategies concerning personalised medicine. By identifying those at greatest risk, the use of immunomodulating therapies could be targeted to prevent progression to disease.

In RA, T cell subset quantification provides an insight into the immune status of the patient.^27^ Although regulatory T cells (Treg) have been the focus of many studies including our own, we have demonstrated that CD4+ T cells are an important T cell biomarker.^7^ ^28^–32 Inflammation causes the cells to differentiate into other subsets driven by proinflammatory cytokines such as interleukin (IL) 6 and tumour necrosis factor (TNF) with the appearance of a novel T cell subset called inflammation related cells (IRCs).^29^ To date, we have demonstrated the role of T cell subset analysis in predicting relapse in DMARD-induced remission,^7^ the safe discontinuation of TNF blockers^31^ and, more recently, methotrexate-induced remission in early RA.^32^

We hypothesised that in ACPA+ individuals with non-specific symptoms, those with the greatest T cell subset dysregulation (as determined using naïve CD4+ T cells, IRC and Treg quantification) would have a greater propensity for progression to arthritis. The aim of this study was to report on the extent of T cell subset dysregulation in ACPA+ individuals and to determine the potential of T cell subset analysis as a biomarker of future progression to clinical arthritis. The confounding effect of clinical parameters previously shown to be predictive in a clinical model^14^ was also investigated.

## Methods

### Patients

As previously described,^14^ individuals with ACPA+ and non-specific musculoskeletal symptoms were identified from regional primary care services and early arthritis clinics. The primary care component was adopted by the UK Primary Care Clinical Research Network (Primary Care Research Network, https://www.crn.nihr.ac.uk/). Individuals 18 years old or over with a new musculoskeletal joint symptom presenting to their primary care physician or health professional were eligible. The following exclusion criteria were applied: fulfilling the European League Against Rheumatism (EULAR) 2010 classification criteria for RA, history of IA diagnosed by a rheumatologist; presence at baseline of clinically detected IA confirmed by a rheumatologist; and use of disease-modifying antirheumatic drugs (DMARDs). ACPA status was determined using the commercially available anti-CCP2 (ImmunoCAP method; Phadia, Sweden, Germany). Eligible participants were recruited to a single centre research clinic as part of a prospective observation cohort. The clinical end point was the development of IA on clinical examination. All participants provided informed consent for the study prior to recruitment. One hundred and six healthy controls provided informed consent for a blood sample draw.

### Clinical assessments

At baseline, demographic details were collected and participants completed patient questionnaires, provided a clinical history of symptoms and had a systems examination by a rheumatologist including a joint count. Individuals attended 3-monthly visits for the 1st year and as clinically indicated thereafter for up to 6.5 years. Participants were able to attend in between visits should they develop any new symptoms. Smoking status was recorded including number of cigarettes smoked and duration. HLA-DRB1 shared epitope (SE) status (low-resolution) was considered positive with the presence of one or two copies of the following alleles: HLA- DRB1*01, DRB1*04 and DRB1*10 in the HLA-DRB1 locus.^33^ ^34^

### T cell subset analysis

Peripheral blood was collected into EDTA. Flow cytometry was performed as previously described.^32^ Briefly, naïve and IRC CD4+ T cell subsets were identified based on their expression of CD45RB-FITC (clone MEM-55, Serotec, Oxford, UK), CD45RA-PE (clone F8-11-13, Serotec), and CD62L-APC (clone 145/15 Coulter, High Wycombe, UK). Treg were quantified by cell surface staining for CD4-Pacifc blue (clone RPA-T4, Bechton Dickson (BD), Oxford, UK), CD25-APC (clone 2A3, BD) and CD127-PE (R34.34, Beckman coulter), followed by intracellular staining for FOXP3-FITC (clone PCH101 eBioscience, San Diego, California, USA) using the antihuman Foxp3 staining kit (Insight Biotechnology, Wembley, UK). Flow cytometry analysis was performed on a LSRII cytometer (BD), using BD Biosciences FACSDIVA software. Subset frequencies were reported as percentage of gated CD3+/CD4+ T cells.

### Statistical analysis

Reference limits for each T cell subset (lower 95% limit of normal for naïve and Treg, upper 95% limit of normal for IRC) were obtained using data from 106 healthy controls (see online supplementary material). We categorised T cell subset values of ACPA+ patients as normal or abnormal according to the lower or upper reference limits, one-sample binomial tests were used to assess whether the proportion of participants with abnormal values differed from the expected 5%. Pearson's χ^2^ tests were used to identify associations between SE and T cell subset abnormalities.

#### Unadjusted associations between T cell subset frequencies and progression to IA

Non-parametrical area under the ROC (AUROC) curve was calculated for each subset. Additionally, sensitivity and specificity of each subset for predicting progression to IA at any time during follow-up were calculated, with 95% CIs estimated by the Wilson method.

#### Adjusted associations between T cell subset frequencies and progression to IA

Binary logistic regression models of the occurrence of progression to IA, and Cox proportional hazards models of time to progression were constructed to adjust for the following variables: age; SE status (negative/positive); smoking (never/ever); ACPA titre. Clinical variables from a previously published model were also included: physician assessed small joint tenderness (absent/present) and duration of early morning stiffness (EMS) (<30 min/≥30 min). Further details on methodology are provided in online supplementary material. Akaike Information Criteria (AIC) values were used to compare the different models (lower AIC indicates a better quality model). The predicted probability of progression obtained from the final logistic regression model (model-3) was calculated for patients with full data; patients were then categorised as being at low (<20%), moderate (20–80%) or high (>80%) risk. Kaplan-Meier plots and log-rank tests were then produced for time to progression, using these risk groups.

Analyses were performed using STATA V.13.1.

## Results

### Reference limit

Samples from 106 healthy controls (HC) enabled the development of reference range for each T cell subset (see online supplementary material). Naïve cells frequency was lower in older HC^29^ but did not differ by gender (see online supplementary material figure S1 and table S1). IRCs were not related to demographic parameters (see online supplementary figure S2). A clear positive association between Treg frequency and age (see online supplementary figure S3 and table S3) and no difference by gender is reported here as recently described.^35^

### Progression to inflammatory arthritis

Of the participants 48/103 (46.6%) developed synovitis during follow-up, with the majority of individuals, 30/48 (62.5%) progressing within 12 months. Baseline clinical and demographic characteristics are presented in table 1.

**Table 1:** Clinical characteristics of ACPA+ individuals (n=103)

Characteristic	Result
Progressed (ever): % (n)	46.6% (48)
Duration of follow-up (months) median (range)	18.4 (0.1 to 79.6)
Age (years) mean (SD; range)	52.6 (11.7; 27 to 79)
Female: % (n)	71.8% (74)
SE positive %(n)*	73.5% (72)
High positive ACPA and/or RF†: % (n)	85.4% (88)
Smoker: % (n)	
Non-smoker	30.1 (31)
Ex-smoker	41.7 (43)
Current smoker	28.2 (29)
EMS≥30 mins: % (n)	34.0% (35)
Small joint symptoms: % (n)	43.7% (45)

*Available in 98/103 patients.

†Determined as >3×upper limit of normal.

ACPA, anticitrullinated protein antibody; EMS, early morning stiffness; RF, rheumatoid factor; SE, shared epitope.

### T cell subset and progression

**Figure 1:**
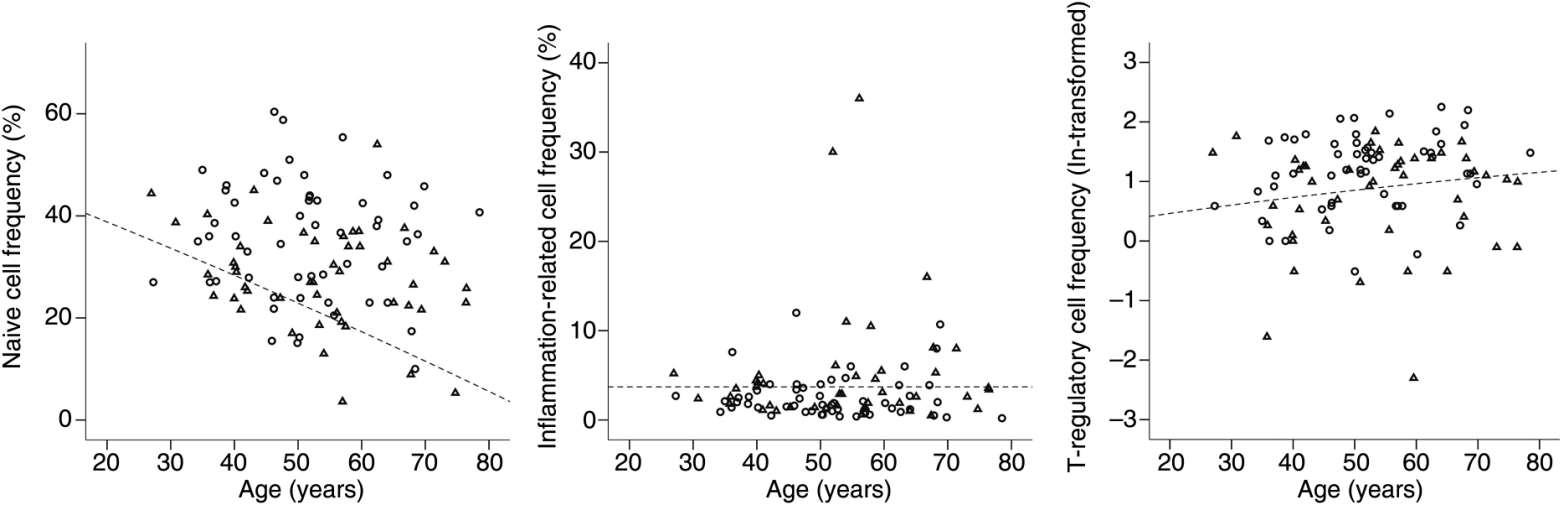
Graphical representation of the T cell abnormalities. T cell subsets were quantified and data presented in relation to the age of the participants. Dotted lines represent the lower limit of normal (LLN) for naive cells and regulatory T cells (Treg) and the upper limit of normal (ULN) for inflammation related cells (IRC).

No abnormal lymphocyte counts were observed in these participants and their CD4+T cells were not different between progressors and non-progressors (data not shown, p=0.794). Frequencies observed for all three subsets are presented in figure 1.

The values observed in ACPA+ individuals were then dichotomised as within or below normal range (defined by the lower limit of normal (LLN) as indicated with the line on figure 1), for naïve T cell and Treg and within or above normal range for IRC (defined by the upper limit of normal (ULN)). In subjects with full T cell data available for all three subsets (n=95/103), a significantly larger proportion of ACPA+ participants had abnormal values (table 2, p<0.001 for all three subsets). A third of ACPA+ individuals had no T cell abnormalities (37.9%), over a third (40.0%) had either one of the three abnormalities and the remaining subjects had two (18.9%) or three (3.2%) abnormalities.

**Table 2:** Unadjusted T cell analysis of progression to IA

	Reduced naïve cell frequency	Elevated IRC frequency	Reduced Treg frequency
Observed proportion of patients (observed/n)	22.5%	30.3%	35.4%
	23/102	30/99	35/99
Calculated proportion*	22.1%	29.5%	35.8%
95% CI	14.2% to 31.8	20.6% to 39.7	26.2% to 46.3
Standardised binomial test z	7.4	10.7	13.5
p Value	<0.001	<0.001	<0.001
AUROC*	0.63	0.63	0.66
95% CI	0.52 to 0.74	0.52 to 0.74	0.55 to 0.77
p Value	0.029	0.032	0.008
Sensitivity*	28.6 %	35.7 %	45.2 %
95% CI	17.2 to 43.6	23.0 to 50.8	31.2 to 60.1
Specificity*	83 %	75.5 %	71.7 %
95% CI	70.8 to 90.8	62.4 to 85.1	58.4 to 82.0
	**Naïve** **(per %)†**	**IRC** **(per %)**	**Treg** **(per %)†**
Unadjusted OR*	0.94	1.15	0.70
95% CI	0.90 to 0.98	1.00 to 1.32	0.56 to 0.89

*In patients with data for all three T cell subsets n=95.

†Adjusted for age.AUROC, area under the ROC curve; IA, inflammatory arthritis; IRC, inflammation related cell; Treg, regulatory T cells.

We observed these patients for up to 5 years before progression to synovitis and obtained a repeat blood sample at an annual visit for 55 of them. Of 26 progressors, 18 participants changed dichotomisation group from normal to below-LLN for naïve or Treg or increase in IRC to above-ULN. Of the 29 non-progressors, only 3 showed changes in dichotomisation.

Using categorised subset values, AUROC analysis confirmed that T cell subsets were predictive individually (table 2). AUROC comprised between 0.63 and 0.66 (p<0.03) with the best individual predictor being Treg, then naïve cells and last IRCs (table 2). Individually, naïve, IRC and Treg subsets also demonstrated high specificities (table 2, 71.7–83.0%) for prediction of progression to IA but had relatively low sensitivities (28.6–45.2%). Consistent with the area under the curve (AUC) analysis, CIs for the unadjusted ORs for all three subsets indicated that, with no adjustment other than age in the case of naïve cells and Treg, each subset was associated with progression to IA (table 2). Higher naïve cell and Treg frequencies were protective, as would be expected, while higher IRC frequencies were associated with increased odds of progression.

### T cell model of progression to IA

Our results suggest associations between T cell subsets and progression; however they could have been confounded by genetic and environmental factors such as SE and smoking.^36–38^ Furthermore, clinical parameters routinely collected in ACPA+ individual were previously used to build a prediction model^14^ and it was important to assess the added value to T cell subset quantification over this initial clinical model. We used regression and compared the performance of three models: a T cell only model (model-1), a clinical only model (model-2) and a combined model (model-3). Details are provided in online supplementary material table S3.

**Figure 2:**
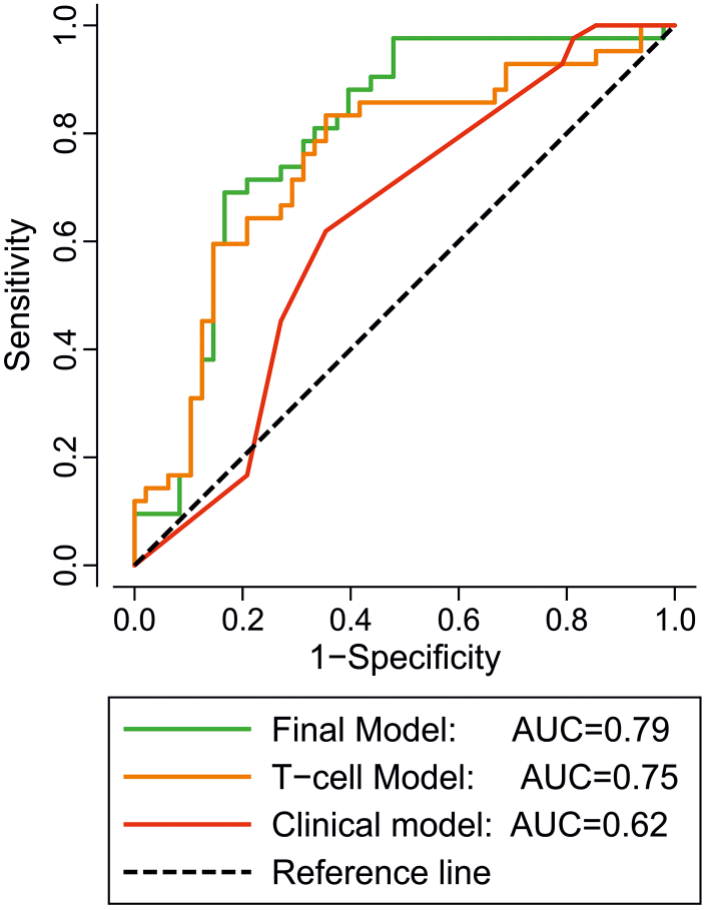
ROC graphical representation of the three logistic regression models. Binary logistic regression models of the occurrence of progression to inflammatory arthritis (IA) using model-1 T cell subset only (orange line), model-2 clinical parameters (red line) and the combined Model-3 (green line) were constructed. The area under the ROC for the predicted probability of progression from model-1 was 0.75 (95% CI 0.65 to 0.85), which represents an improvement over model-2 (0.62 (0.54 to 0.76)). Model-3 showed the best results with an area under the ROC at 0.79 (0.79 to 0.89).

AUROC curves were constructed for all three models (figure 2). Although the AUROC curve for the combined model (model-3 AUC 0.79) was better than for the T cell only model (model-1 AUC 0.75), the AIC values suggested that the combined model-3 did not represent a major improvement over the T cell only model-1 (see online supplementary table S3, 116.3 vs 115.7), but it did offer added predictive value over the clinical model-2 (AIC 116.3 vs 125.0). Both of these adjusted models improved on the values achieved by looking at each subset individually (table 2, AUCs comprised between 0.63 and 0.662).

Applying model-3, we then stratified patients into three groups according to their predicted risk of progression: low (0–19%, n=20), moderate (20–79%, n=56) or high (80–100%, n=12) to examine its potential clinical utility. The majority of individuals in the high-risk group progressed (64%, 9/12) compared with those in the low-risk (5%, 1/20) and moderate-risk (57%) groups.

### Time to progression to IA

Using the three logistic regression models, Cox regression was constructed to investigate time to progression as it has more relevance clinically (see online supplementary table S4). The trends identified were similar. All three T cell subsets showed signs of association with the odds of progression in model-1 and model-3 (see online supplementary table S4, HR), however, IRCs were the most significant in this analysis. Using Harrell's C as an indication of performance of the Cox regression, the combined model-3 allowed 69% of randomly chosen pairs of progression times to be correctly ordered compared with 65% for the T cell only model-1 and 60% in the clinical only model-2 (see online supplementary table S4).

**Figure 3:**
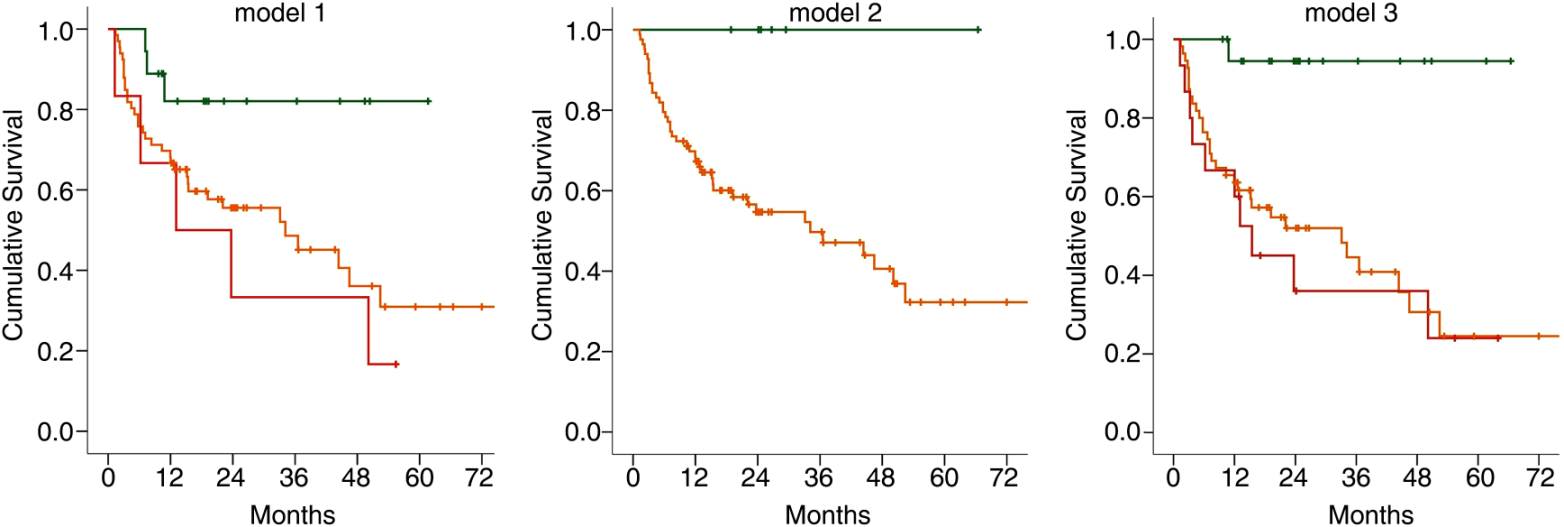
Kaplan-Meier graph of cumulative inflammatory arthritis (IA)-free survival according to predicted probability of progression in the three models. Anticitrullinated protein antibody (ACPA)+ subjects were stratified into three groups according to their predicted risk of progression: low (green line, 0–19%), moderate (orange line, 20–79%), high (red line, 80-100%) calculated for all three models. Kaplan-Meier plots for time to progression were built according to the predicted risk categories from logistic regression model-1 and model-3. Time to progression differed significantly according to risk of progression, in model-1 (χ^2^=6.04, p=0.049) and model-3 (χ^2^=13.43, p=0.001), although there was little difference between the curves for patients in the moderate-risk or high-risk groups in both models. For model-2, the median time to progression in the moderate-risk group is 34.1 months with an overall significant difference between the two risk groups (χ^2^=4.60, p=0.032).

Figure 3 presents Kaplan-Meier plots for time to progression according to the predicted risk categories from logistic regression for the three models. Time to progression differed significantly according to risk groups in model-1 (χ^2^=6.04, p=0.049) and model-3 (χ^2^=13.43, p=0.001), although there seemed to be little difference between the curves for patients at moderate risk or high risk of progression. For model-2, none of the patients at low risk progressed so we could not compute time to progression for that group. The median time to progression in the moderate-risk group was 34.1 months with an overall significant difference between the groups (χ^2^=4.60, p=0.032). In model-3, those within the high-risk group progressed to IA much more rapidly (median 15.4 months, (95% CI 14.3 to 40.8)) compared with those in the moderate-risk group (35.1 months (25.8–44.4)) and the low-risk group (63.4 months (57.9–69.3)).

## Discussion

This is the first analysis exploring T cell subset quantification within a prospectively followed ‘at-risk’ population. We demonstrated that T cell subsets dysregulation predates the onset of IA in ACPA+ individuals. Compared with healthy controls, two-thirds of the ACPA+ participants demonstrated T cell abnormalities in one or more of the three subsets analysed, these subsets being individually predictive (table 2). Furthermore, the potential use of these biomarkers within an exploratory prediction model is presented. Adjusted analysis demonstrated the added value of combining T cell subsets and clinical data (figure 2 and see online supplementary table S3) and the time to progression was significantly reduced with increasing risk of progression (figure 3, see online supplementary table S4).

Individuals with systemic autoimmunity and musculoskeletal (MSK) symptoms (+/−arthralgia) but no clinical synovitis (groups c+d using EULAR terminology^8^) have been studied prospectively in several research centres.^10^ ^14^ Although the suggested terminology has assisted in classifying individuals there remains great heterogeneity considering the possible genetic predisposition, environmental factors, symptomology, and ultimately progression or not towards arthritis reported in these studies. This limits the transferability of findings between studied populations, however, the information gained offers a unique opportunity to understand possible ‘at-risk’ states and triggers. Although 53.4% (55/103) of our subjects have yet to develop IA, some progressed rapidly—these individuals should benefit from early targeted therapies. Identifying these individuals is therefore a clinical priority. One group of interest would be those with two T cell abnormalities and a high specificity (but low sensitivity) for progression. However it may be equally important to be able to identify and reassure those who are at little risk.

The positive and negative predictive values of the combined model-3 were 60% (41/68) and 95% (19/20), respectively. While a positive predictive value (PPV) of 60% is lower than clinically desired, it is an improvement on the T cell model-1 and clinical model-2 (54% and 50%, respectively). Furthermore the negative predictive value (NPV) of 95% of the combined model-3 suggests that it is possible to predict quite accurately which participants are unlikely to progress and who may therefore be safely discharged. Compared with the clinical model-2 which is best at predicting non-progression (NPV 100%) this suggests that adding T cell data to clinical data clearly improved the ability to determine progressors. Reviewing data from annual visits further strengthened the concept of T cell abnormalities developing over time, and given that progression may happen up to 5 years later it may not be that unexpected that PPV is 60% at baseline. This supports the need for annual review in the moderate-risk (and high-risk) group with re-evaluation of the risk score and tailoring of clinical care.

Several other biomarkers of progression have been considered including imaging modalities, possible pathogens and several immune-mediated markers.^16^ ^19^ ^25^ ^26^ ^39–41^ Although a specific cytokine profile could not be associated with progression,^41^ the hypothesis of a mounting immune response prior to IA development has been supported by reports of a broadening of the repertoire of citrullinated peptides associated with imminent progression to IA^19^ as well as a possible IFN-type-1 gene expression signature.^26^ There are limited studies investigating T cell subsets within an ‘at-risk’ population. We have demonstrated that all three subsets are individually associated with progression. Although all three subsets are part of the CD4+ T cell pool, they are generated through different mechanisms and their individual frequencies are not related to one another. The development of IRCs is the result of inflammatory processes whereas naïve cells are generated in the thymus and released to circulate in the blood. Naturally occurring Treg also develop in the thymus, however, their frequency in the blood is not directly related to thymic release.^42^ Therefore the individual status of all three subsets reflects the immunological state of an individual at a particular point in time (ie, a biomarker). A small cross-sectional study of 26 seropositive patients with arthralgia reported on peripheral naïve T cells (CD3+CD4+CD45RO−CCR7+),^43^ however, no difference between health, RA and arthralgia was reported in this study. Naïve CD4+ T cells are conventionally identified using markers such as CD45RA+, CD62L+ and CD44+, but less often using an exclusion marker (CD45RO−) and an activation marker (CD197/CCR7). It is therefore difficult to compare these data with our findings; also, given the use of a mixed cohort of ACPA+ and/or IgM-FR+ patients with arthralgia, several studies having showed clear reduction of naïve cells and Treg in early RA.^28^ ^30–32^ Loss of immune regulation may be an important immunological event in the progression of a disease. However, at this stage, we cannot establish that one of the abnormalities is ‘more important’ than another. Our data suggest that all three are contributing to the risk, as the modelling demonstrates independent predictive value, each possibly reflecting a different aspect of RA pathogenesis.

To date, flow cytometry in patients with RA has been predominately performed in a research setting, however we have established a pathway within our local National Health Service (NHS) services to make the test routinely available and the data presented were mostly generated using this facility. It is hoped that this approach will offer robust sample processing and capacity to allow for validation of these results. To be of significant clinical utility, it will be necessary to identify patients at high risk of progression over a known (and ideally short) period of time. Replication of the current findings will enable this to occur.

Our current model produces a risk prediction per % of naïve cell, Treg or IRC lost or gained, therefore relating the strength of the prediction to the levels of numerical T cell abnormalities. However, defining a threshold level of abnormality would be more practical for a risk score. The current and previously published clinical models^14^ were pilot analyses providing preliminary biomarkers. In this study, clinical predictors performed either as well (strong positive ACPA), worse (EMS) or better (small joint involvement) than previously, with the overall result that the clinical model-2 is performing slightly less well. This is not unexpected as predictive models are known to suffer from coefficient shrinkage.^44^ Nevertheless, the added value of combining T cell data with clinical parameters is clearly demonstrated. Our plan is to finalise the combined models in a larger group of participants, developing rules for clinical application and including a threshold for T cell abnormalities.

## Supplementary Material

Web supplement
