# Supplementary material for: T cell subsets: an immunological biomarker to predict progression to clinical arthritis in ACPA-positive individuals
Source: Ann Rheum Dis. 2015 Dec 1;75(10):1884–9. doi: 10.1136/annrheumdis-2015-207991 (PMC5036223; doi:10.1136/annrheumdis-2015-207991)
Supplement: Web supplement [file annrheumdis-2015-207991-s1.pdf]

Supplementary Material

T-cell subsets: An immunological biomarker to predict progression to clinical arthritis in ACPA positive individuals.

Authors : L Hunt, EM Hensor , J Nam, A Burska, R Parmar, P Emery, F Ponchel.

Address: LIRMM and LMBRU

Corresponding author: PE

Keywords: at risk ACPA+ individual, naïve T-cells, regulatory T-cells,

Reference limits for T-cell subsets

Multiple linear regression was used to assess whether T-cell subset frequencies varied by age or sex. Where associations with age were found, one-sided 95% prediction intervals for the association were obtained by calculating two-sided 90% intervals and discarding the upper or lower interval accordingly; a 90% confidence interval around the prediction interval was calculated. Otherwise, the 5% or 95% centile, estimated assuming a normal distribution, and robust 90% confidence interval around it were calculated. T-cell subset frequencies found to be skewed were ln-transformed prior to analysis. Back-transforming to the original units yielded asymmetric confidence intervals.

<u>*Naïve cells*</u>

There was no evidence that naïve cell frequency differed between males and females [age-adjusted difference (95% CI) -1.44% (-4.77%, 1.89%); p=0.392], or that its association with age differed by sex [difference in slope -0.02% (-0.24%, 0.27%) per year; p=0.910], but there was a highly statistically significant tendency for naïve cell frequency to be lower in older people [slope -0.54% (-0.67%, -0.42%) per year; p<0.001]. The reference limit was therefore adjusted for age but was not stratified by sex. Naïve cell frequency was available for 106 controls; mean (SD) age 43.54 (12.52), range 19 to 69.

Supplementary Figure 1S: Scatter plot of naïve cell frequency (%) and age.

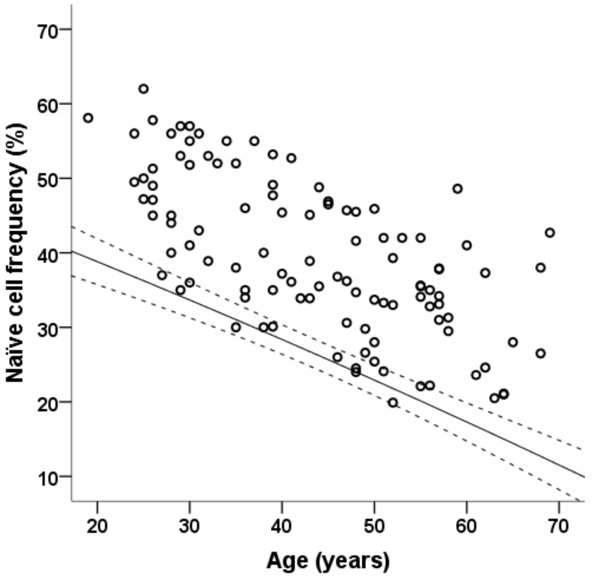

Solid line = Lower limit of normal, Dashed line = 90% CI

To calculate a one-sided 95% prediction interval, a two-sided 90% interval was calculated and the upper limit discarded.

The lower reference limit and its 90% confidence interval were calculated to be

(reference limit)

$$b_0 + (b_1 \times age) - 1.645 \times S \times a_{n-p}$$

(90% CI)

$$\pm 1.645 \times a_{n-p} \times S \times \left(v + 1.645^2\left(a_{n-p}^2 - 1\right)\right)^{1/2}$$

where S is the root mean square error, $a_{n-p} = ((n-p)/(n-p-0.5))^2$, $v = (1/n + (x_0 - \bar{x})^2/\sum_i(x_i - \bar{x})^2)$ and $\bar{x}$ is the mean age.

The naïve lower limit of normal and corresponding 90% confidence interval around it were calculated as LLN = ((-0.54*x)+63.19)-(1.645*sqrt(62.03)*(1+(1/106)+(((x-43.54)*(x-43.54))/16460.35)))±1.645*sqrt(62.03)*(((106-2)/(106-2-0.5))**0.5)*((((1/106)+(((x-43.54)*(x-43.54))/16460.35))+((1.645*1.645)*(((106-2)/(106-2-0.5))-1)))**0.5)

Supplementary Table 1S: Lower limit of normal naïve cell frequency for ages in the range 25 to 65 years

Age	Lower limit of normal (90% CI)
25	36 (34, 39)
30	34 (31, 36)
35	31 (29, 33)
40	28 (26, 30)
45	26 (24, 28)
50	23 (21, 25)
55	20 (18, 22)
60	17 (15, 20)
65	14 (12, 17)

Outside this range of ages it is possible that the association between age and naïve cell frequency may not be linear; therefore, until more data are collected for controls aged under 25 or over 65 are collected, the lower limit for these age groups will not be provided.

Inflammation related cells (IRC)

Data were ln-transformed prior to analysis. There was no evidence that IRC differed between males and females [geometric mean ratio 0.98 (0.68, 1.41); p=0.900] or varied with age [change -0.41% (-1.73%, 0.93%) per year; p=0.544]. IRC frequency was available for 101 controls; mean (SD) age 43.50 (12.69), range 19 to 69. The 95% centile and its 90% confidence interval calculated using a robust method (back-transformed to original units) were 3.70 (3.30, 7.00), corresponding to the upper limit of normal for IRC.

Supplementary Figure 2S Scatter plot of IRC (%) and age.

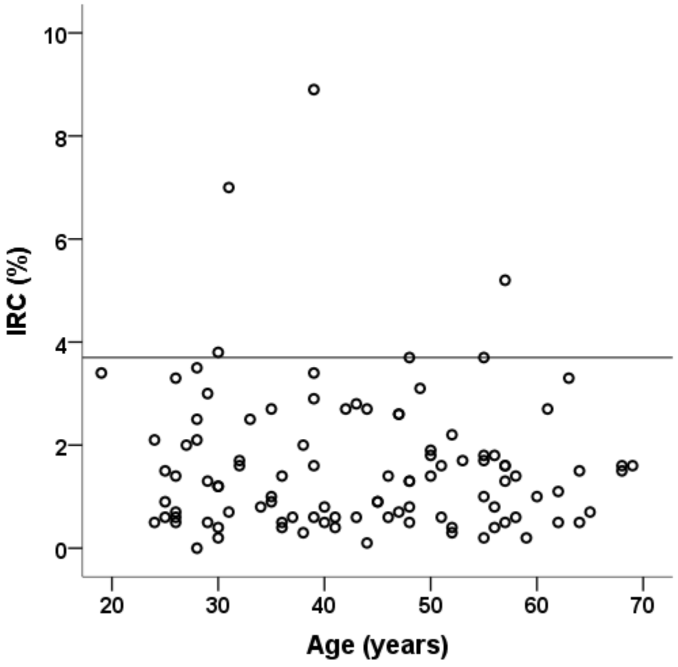

T-regulatory cells (Tregs)

Data were ln-transformed prior to analysis. There was no evidence that IRC differed between males and females [geometric mean ratio 0.98 (0.68, 1.41); p=0.900] or varied with age [change -0.41% (-1.73%, 0.93%) per year; p=0.544]. IRC frequency was available for 101 controls; mean (SD) age 43.50 (12.69), range 19 to 69. The 95% centile and its 90% confidence interval calculated using a robust method (back-transformed to original units) were 3.70 (3.30, 7.00), corresponding to the upper limit of normal for IRC.

Supplementary Figure 1S: Scatter plot of ln-transformed Treg frequency (%) and age

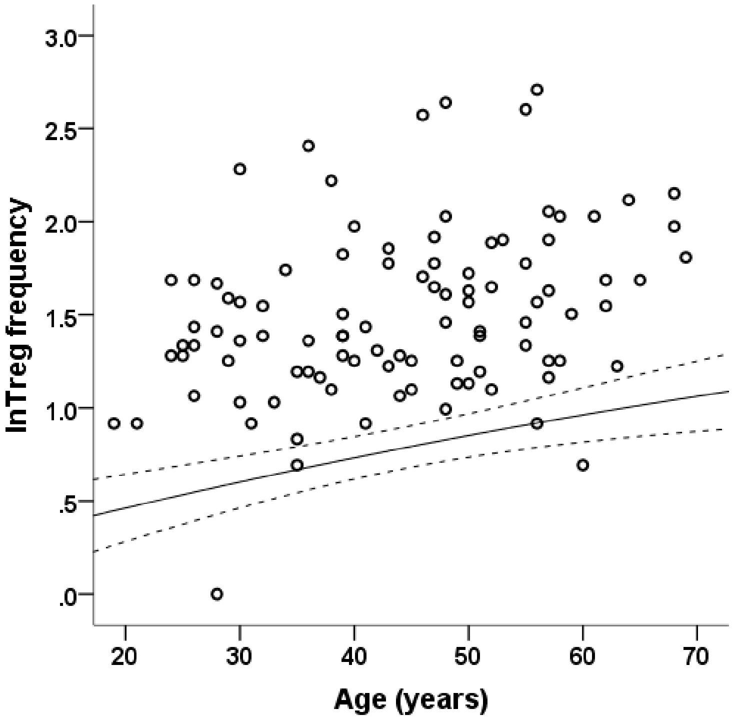

(Solid line = Lower limit of normal, Dashed line = 90% CI)

There was no evidence that T-regulatory cell frequency differed between males and females [age-adjusted geometric mean ratio 1.03 (0.82, 1.22); p=0.976], or that its association with age differed by sex [ratio of differences in slope 1.00 (0.99, 1.02); p=0.677], but there was a statistically significant tendency for Treg cell frequency to be higher in older individuals [by 1.22% (0.50%, 1.94%) per year; p=0.001]. The reference range was therefore adjusted for age but was not stratified by sex. Treg cell frequency was available for 98 controls; mean (SD) age 44.09 (12.30), range 19 to 69.

The Treg lower limit of normal and corresponding 90% confidence interval around it were calculated as = ((0.01*x)+0.97)-(1.645*sqrt(0.19)*(1+(1/98)+(((x-44.09)*(x-44.09))/14670.17))+1.645*sqrt(0.19)*(((98-2)/(98-2-0.5))**0.5)*((((1/98)+(((x-44.09)*(x-44.09))/14670.17))+((1.645*1.645)*(((98-2)/(98-2-0.5))-1)))**0.5)

Supplementary Table 2S: Lower limit of normal Treg cell frequency for ages in the range 25 to 65 years

Age	Lower limit of normal (90% CI)
25	1.71 (1.46, 2.00)
30	1.83 (1.59, 2.10)
35	1.95 (1.72, 2.21)
40	2.08 (1.86, 2.33)
45	2.21 (1.98, 2.47)
50	2.34 (2.09, 2.63)
55	2.48 (2.18, 2.82)
60	2.62 (2.26, 3.03)
65	2.76 (2.33, 3.25)

T-cell Model of Progression to inflammatory arthritis

Binary logistic regression models of the occurrence of progression to IA, and Cox proportional hazards Models of time to progression were constructed. Models were produced sequentially to investigate the effects of adding in covariates. Having obtained unadjusted odds ratio estimates, firstly an adjusted model containing only the T-cell subsets and age was specified (model-1). We then compared results for the variables from the published clinical model (model-2) to a Model that added in the T-cell pathway (model-3). Analyses were first performed in the subset of patients with full data to permit model performance to be tested. Link tests were performed to check for specification error in the logistic regression Models and Hosmer and Lemeshow goodness-of-fit tests were performed. Concordance was assessed for Cox regression models and the proportional hazards assumption was tested. To account for missing data, multiple imputation using chained equations was then used to produce 20 complete datasets, the results from which were combined according to Rubin's rules.

Intermediate models were first constructed to investigate the effect of genetic (SE) and environment (smoking) and to build the final model where some clinical parameters had to be eliminated to fit the limitation imposed by our relatively small samples size.

Model-1: When all three subsets were included in a model with age (Figure 2 and Table 5S), naïve and Treg were independently associated with progression notably compared to the unadjusted OR (Table 4S), while the effect of IRC was less prominent. The area under the ROC for the predicted probability of progression from this model was 0.75 (95%CI 0.65, 0.85), which represents an improvement over the prediction by the 3 subsets individually (Table 4S).

Model-2: The clinical model consisted of antibody status (RF and/or ACPA titre 3x the upper limit of normal), EMS >30 minutes and physician assessed small joint symptoms[14]. Within this patient group (n=95), EMS was not independently associated with the odds of progression to IA in this group of patients (Table 5S, p=0.997) but autoantibodies status and the presence of small joint symptoms were (Table 5S, p=0.026 and p=0.024 respectively). The area under the ROC for model-2 was 0.62 (0.54, 0.76)

Model-3: Adding the T-cell subsets to the clinical model was challenging because the sample size was relatively small for the number of variables to be used[37]. In such cases it is recommended that the least significant of the variables in the full model are removed, provided this does not substantially affect the ORs for the remaining variables[38]. When the

variables from model-1 and 2 were combined also considering SE and smoking, EMS (p=0.553) and smoking (p=0.627) were the least significant and were therefore removed. Age was retained (p=0.668) because its removal affected the ORs for naïve and Treg. Having adjusted for age, SE, autoantibody status and joint counts, naïve and Treg frequencies remained independently associated with the odds of progression (Table 5S, p=0.008 and p=0.015 respectively) but no longer IRC (p=0.441). The area under the ROC was 0.79 (0.70, 0.89), which improved compared to model-1 showing the added value of combining both data sets.

By constructing sequential logistic regression models we have demonstrated the potential for clinical utility of combining biomarkers. Here, we first demonstrated the value of using T-cell subsets together in the same model (model-1) compared to using each subset individually. Secondly we showed that T-cell subsets remained independently associated with the odds of progression, even when adjusting for clinical, genetic and environmental factors (model-3). The AIC is a measure that trades off the information a model provides about the outcome against its complexity; on the basis of the AIC values the final adjusted model (model-3) was not offering major improvement over the T-cell model (model-1), although the AUC ROC was slightly better. The survival analyses further support the hypothesis that, those with the greatest T-cell subset dysregulation are at the greatest risk of imminent progression. The clinical need to review closely those individuals considered at high risk would be paramount while those in the low risk group could be seen less frequently or discharged with a view to repeating T-cell subsets and clinical examination at a later point (maybe yearly).

Although this is a relatively large study of ACPA+ at risk individuals, the sample size has limited the robustness of statistical Modelling. It is recommended that there should be at least 10 cases in the smallest outcome category (‘events’) per variable (EPV), although it has been shown that valid results can be obtained with EPVs between 5 and 9 provided the results are interpreted cautiously. In Model-3 the EPV was 6.9, therefore we feel that these are promising preliminary results, but this Model must be considered exploratory until it is validated in a second cohort.

Table 3S : Logistic regression models of progression to IA.

Covariates		Model-1	Model-2	Model-3
Naive (per %)*	OR<br>95% CI<br>p	0.93<br>0.89, 0.97<br>0.002		0.94<br>0.89, 0.98<br>0.008
IRC (per %)*	OR<br>95% CI<br>p	1.07<br>0.94, 1.23<br>0.294		1.05<br>0.92, 1.20<br>0.441
Treg (per %)*	OR<br>95% CI<br>p	0.68<br>0.53, 0.88<br>0.003		0.72<br>0.55, 0.94<br>0.015
Age (per year)	OR<br>95% CI<br>p	1.01<br>0.97, 1.05<br>0.492		1.01<br>0.97, 1.05<br>0.668
SE positive(%)	OR<br>95% CI<br>p			2.36<br>0.76, 7.36<br>0.138
Smoker				removed[†]
High positive RF /ACPA[&]	OR<br>95% CI<br>P		4.66<br>1.21, 18.05<br>0.026	2.79<br>0.58, 13.34<br>0.198
Small joint symptoms	OR<br>95% CI<br>p		2.65<br>1.14, 6.19<br>0.024	2.14<br>0.84, 5.46<br>0.110
EMS ≥30 mins	OR<br>95% CI<br>p		1.00<br>0.41, 2.42<br>0.997	removed[†]
Model Properties				
Goodness of fit p		0.408	0.070	0.128
Pseudo-R^2		0.15	0.06	0.19
AIC		115.7	125.0	116.3
AUROC<br>95% CI		0.75<br>0.65, 0.86	0.62<br>0.54, 0.76	0.79<br>0.70, 0.89

* adjusted for age, AIC=Akaike information criterion; AUROC=area under the ROC curve; & determined as >3XULN=upper limit of normal [†]removed from final Model to reduce the number of covariates.

Table 4S : Results of Cox regression models of time to progression to IA.

**Covariates**		**Model-1**	**Model-2**	**Model-3**
Naive (per %)*	HZ	0.97		0.97
	95% CI	0.94, 0.99		0.95, 1.00
	p	0.018		0.044
IRC (per %)	HZ	1.08		1.08
	95% CI	1.02, 1.15		1.01, 1.15
	p	0.006		0.016
Treg (per %)*	HZ	0.83		0.86
	95% CI	0.70, 0.98		0.72, 1.02
	p	0.027		0.091
Age (per year)	HZ	1.01		1.00
	95% CI	0.98, 1.03		0.98, 1.03
	p	0.598		0.791
SE positive	HZ			1.60
	95% CI			0.66, 3.86
	p			0.297
Smoker				not entered[†]
High positive RF / ACPA[&]	HZ		2.44	1.45
	95% CI		0.75, 7.92	0.41, 5.08
	p		0.139	0.561
Small joint symptoms	HZ		1.73	1.54
	95% CI		0.96, 3.12	0.86, 2.77
	p		0.071	0.149
EMS ≥30 mins	HZ		1.21	not entered[†]
	95% CI		0.66, 2.21	
	p		0.536	
Model properties				
Harrell's C		0.65	0.60	0.69
AIC		329.6	335.7	332.4

* adjusted for age, AIC=Akaike information criterion; AUROC=area under the ROC curve; & determined as >3XULN=upper limit of normal [†]removed from final Model to reduce the number of covariates. HR=hazard ratio.
